# Fusion of Haptic and Gesture Sensors for Rehabilitation of Bimanual Coordination and Dexterous Manipulation

**DOI:** 10.3390/s16030395

**Published:** 2016-03-18

**Authors:** Ningbo Yu, Chang Xu, Huanshuai Li, Kui Wang, Liancheng Wang, Jingtai Liu

**Affiliations:** 1 Institute of Robotics and Automatic Information Systems, Nankai University, Tianjin 300353, China; xuch@mail.nankai.edu.cn (C.X.); lihuanshuai@mail.nankai.edu.cn (H.L.); wangkui337@mail.nankai.edu.cn (K.W.); liujt@nankai.edu.cn (J.L.); 2 Tianjin Key Laboratory of Intelligent Robotics, Nankai University, Tianjin 300353, China; 3 Rehabilitation Center, Tianjin Hospital, Tianjin 300211, China; cmoweb@126.com

**Keywords:** haptics, rehabilitation, bimanual coordination, dexterous manipulation, sensory-motor cognitive skills

## Abstract

Disabilities after neural injury, such as stroke, bring tremendous burden to patients, families and society. Besides the conventional constrained-induced training with a paretic arm, bilateral rehabilitation training involves both the ipsilateral and contralateral sides of the neural injury, fitting well with the fact that both arms are needed in common activities of daily living (ADLs), and can promote good functional recovery. In this work, the fusion of a gesture sensor and a haptic sensor with force feedback capabilities has enabled a bilateral rehabilitation training therapy. The Leap Motion gesture sensor detects the motion of the healthy hand, and the omega.7 device can detect and assist the paretic hand, according to the designed cooperative task paradigm, as much as needed, with active force feedback to accomplish the manipulation task. A virtual scenario has been built up, and the motion and force data facilitate instantaneous visual and audio feedback, as well as further analysis of the functional capabilities of the patient. This task-oriented bimanual training paradigm recruits the sensory, motor and cognitive aspects of the patient into one loop, encourages the active involvement of the patients into rehabilitation training, strengthens the cooperation of both the healthy and impaired hands, challenges the dexterous manipulation capability of the paretic hand, suits easy of use at home or centralized institutions and, thus, promises effective potentials for rehabilitation training.

## 1. Introduction

Neural impairment, such as stroke, is the most common reason bringing tremendous physical and mental burden to patients, families and society [1,2]. Each year, the new incidence of stroke affects 15 million people worldwide, and the incidence rate doubles for every decade after 55 years of age [3,4]. The dominant functional disabilities in about 80% of patients with stroke are partial upper extremity paralysis and deficits in motor control capabilities, consequently causing limitations in executing the activities of daily living (ADLs) [5,6,7,8]. Grounded on neuroplasticity and supported by motor learning theories, neurorehabilitation therapy helps patients to partially or fully restore the lost functions, bringing promising recovery, more independent living and better social involvement [4,9,10,11,12].

Two different types of upper extremity rehabilitation methods have been developed complying with the neurorehabilitation principles: unilateral and bilateral arm training [13]. Unilateral arm training represents the conventional constrained-induced therapy, emphasizing massive training on the paretic arm and constraint on the healthy arm to restrict neural compensation in the meantime [1,14,15]. However, humans are inherent to perform bilateral movements and coordinate the motion between both sides of their bodies. A person can easily move both hands simultaneously to produce concerted actions [16]. Besides, bimanual skills are frequently required for bilateral symmetrical tasks in ADLs, including basic self-care skills, fundamental mobility functions and instrumental activities [17]. Thus, coupled bilateral re-training is essential and also promising for patients with severe arm impairment. Compared to the conventional unilateral rehabilitation method, bilateral skills require a different neuromotor control mechanism, while strong behavioral coupling exists in the simultaneous movements [18,19,20]. For the ultimate goal of regaining bimanual motor capabilities, the unilateral training method is, on the other hand, not optimal, due to differences in the neurophysiological and behavioral mechanisms; bilateral re-training is necessary for stroke patients and can achieve at least partial recovery on unilateral skills [7,13,17]. In view of the bilateral nature of the therapy, the subjects are encouraged to coordinate both hands by allowing the intact arm to dominate the therapy of the paretic arm and extending performable range to re-learn bilateral skills through meaningful bimanual interaction [15,16]. Both the ipsilateral and the contralateral sides of the neural injury are actively involved during the bimanual interaction, which promotes the cooperation of both sides of the central nervous system and makes it easier for patients to restore bimanual coordination than unilateral training. Since most of the assistance comes from the patient, external assistance is less required from powerful motors or a caregiver [1,3].

In the past few years, many robot-assisted bilateral rehabilitation devices have been developed, specifically for providing synchronous arm training for ADLs. Bilateral symmetrical motions, such as mirror motions, are biomechanically interesting because the efferent signals can be duplicated at a low level, where the joints on each limb are identical [16]. The Mirror Image Movement Enabler (MIME) has been designed to coordinate bimanual trajectory in a mirror symmetry manner [21]. The robot continuously moves the paretic arm to the mirror position and orientation of the intact arm. An improvement in proximal mobile capabilities and muscle strength of the impaired arm has been obtained [17,22]. The Bi-Manu-Track system, designed for synchronous bimanual motion, enables duplicate practice for mirror movements of wrist flexion-extension and forearm pro-supination. The result shows that the bilateral training approach can improve motor function in different neurophysiological mechanisms [13,23,24]. Bilateral arm training with rhythmic auditory cuing (BATRAC), based on motor learning principles, has proved to have positive effects on the recovery of upper extremities for stroke survivors [18]. Further, the ideal physical parameters for coupled symmetric interaction have been explored via a compliant bimanual rehabilitation device (CBRD) [25].

The bimanual symmetry mechanism has been extensively explored through the rehabilitation devices mentioned above. However, there are relatively few mirrored bilateral motions required in ADLs. Common bilateral symmetrical modes include visual symmetry, point mirror symmetry in task space and joint space symmetry [16]. All of these symmetric modes share almost uniform functional parameters, and both arms anticipate stable patterns, making it easier for the neural system to react and tricky for utilizing bilateral superiority on coupled interactions [17].

Bilateral complementary motions, other than symmetric motions, are mostly involved in ADLs, such as making knots, unscrewing caps, clipping nails and even ripping packs [15]. Clearly, bilaterally-coordinated cooperation is essential for performing complementary tasks, with each arm having a unique functional role that requires simultaneous asymmetric motor control capabilities [15,17]. Given the apparent significance of bilateral complementary interactions in ADLs, systematic studies are needed to recognize, re-train and assess the supportive function, as well as the dominant role of the paretic arm. Furthermore, robot-assisted bilateral complementary training should be developed and evaluated.

The positive effects of virtual reality on rehabilitation therapy have been extensively validated, and impaired motor capabilities are improved via robot-assisted sensorimotor activities [1,26,27]. More engaging therapies, ensured safety and deep immersion can be obtained with force feedback and integrated into the designed virtual scenario and rehabilitation sessions. In our previous work [4], a *“flying through a spatial tunnel”* scenario has been realized with a haptic interface to re-train the dexterous manipulation capabilities of the upper limb. The bimanual haptic desktop system (BHDS) integrated with the video display terminal (VDT) has been established for video game-like exercises in a bimanual manner [28].

In this paper, a novel task-oriented method is proposed for bilateral rehabilitation training, focusing on the cooperation of both the upper limbs and the dexterous manipulation of the impaired limb. The training paradigm has been implemented with the fusion of the Leap Motion gesture sensor and the omega.7 haptic sensor with force feedback capabilities. A virtual scenario for the bimanual training tasks is designed and built up. The system is compact, easy to install and transfer. Thus, it suits convenient use at home, as well as in centralized rehabilitation training institutions. Preliminary experiments with healthy subjects have validated the feasibility of the proposed bilateral rehabilitation training system and method.

## 2. Methods

### 2.1. Design of the Bimanual Complementary Tasks and Virtual Scenario

Extensively involved in ADLs, bilateral complementary coordination is classified as the most common type of bilateral skill, where two arms are coordinated in cooperation, with each having a unique functional role, but essentially simultaneous temporal, spatial and force parameters. Compared to the symmetric bimanual movements, significant different motor control capabilities are required from each arm in a complementary manner [17]. Typical bilateral complementary tasks, such as making knots, tying shoelaces, clipping nails and unscrewing caps, all involve holding an object with the supportive hand and manipulating it with the other dominant hand.

**Figure 1:**
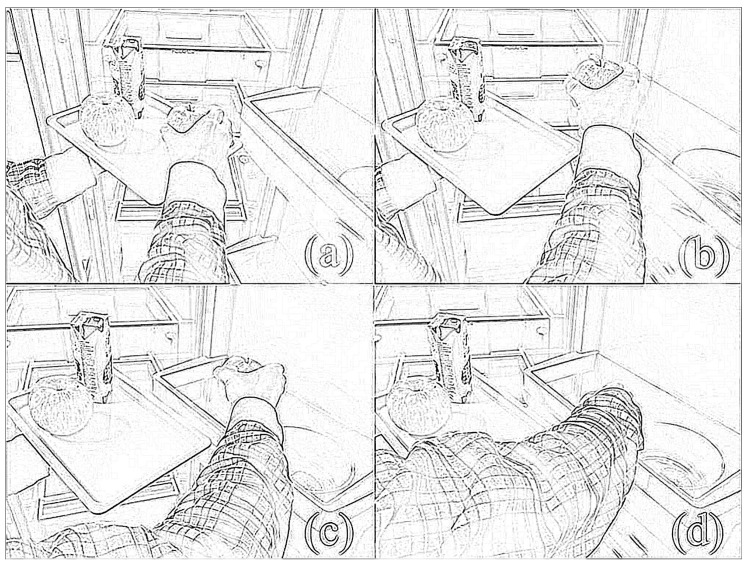
Schematic description of the steps to perform the bimanual complementary task. The left hand, assumed healthy, plays the supportive role and holds the plate. The right hand, assumed impaired, plays the dominant role and performs the task in four steps: (**a**) reaches for the object and holds it; (**b**) takes the object off the plate; (**c**) moves the object towards the target position; (**d**) places the object at the target position.

We design our bilateral complementary training task in the same principle, as shown in Figure 1. The healthy left hand, performing the supportive role, holds and moves the plate actively and cooperatively, to compensate the motion of the right hand. On the other side, the impaired right hand, performing the dominant role, approaches and grasps the specific objects on the plate one by one and puts them onto the corresponding target position steadily and smoothly. Dexterous manipulation is involved and re-trained in the grasp movement, including flexion-extension, radio-ulnar deviation of the wrist joint and pro-supination of the forearm. To conduct the proposed bilateral complementary tasks, the subjects need to actively expand the performable range of their right arm to grasp the objects and put them onto the targeting position, through relearning bilateral coordinated cooperation in a natural way.

**Figure 2:**
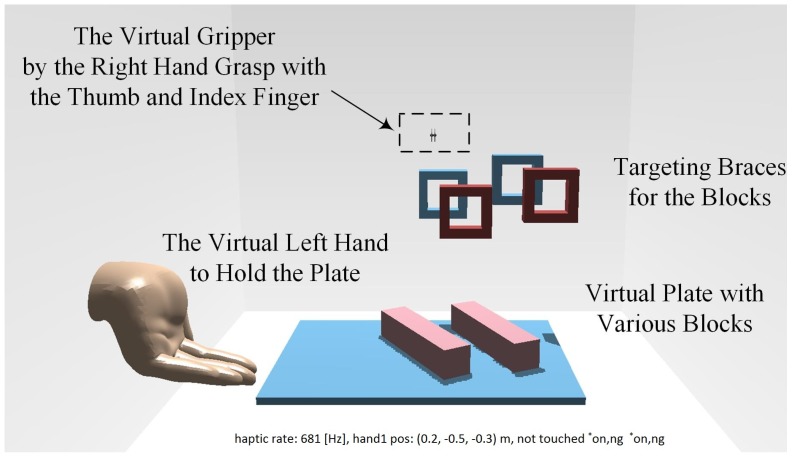
The virtual scenario for the designed bilateral cooperative manipulation task.

A virtual scenario for the bilateral complementary tasks in Figure 1 has been designed and realized as shown in Figure 2. It is based on the CHAI3D haptic engine, an open-source multi-platform framework. As in our previous work [4], CHAI3D can enable real-time simulation for both computer haptics and visual rendering of virtual objects. In addition, CHAI3D supports the omega.7 haptic device well, offering a variety of classes and algorithms that can be developed extensively.

The interaction force in the scenario, as well as the customized friction between the plate, blocks and braces enable dexterous haptic manipulation of the paretic arm. Based on the designed complementary tasks, the training of the active coordinated cooperation of both the paretic arm and intact arm is engaged, while the dexterous manipulation and fine motor control capabilities are particularly trained as required by the grip-reach-release task for the impaired hand.

Fore feedback engaged in the virtual scenario simulation is essential for immersion and the meaningful interaction required by intensive therapy. The force algorithms offered by CHAI3D include the finger-proxy model, potential fields, Coulomb friction models and multi-point contact. To generate haptic perception, all of the virtual objects are modeled as rigid bodies in 3D MAX, along with alternative physical properties, and then imported into the simulation environment. Contact detection complies with the axis-aligned bounding box hierarchies for physical rendering.

To balance the performance and efficiency of the proposed algorithm, the collision detector from Open Dynamic Engine (ODE) has been employed for high speed dynamic calculations and physical rendering, so that the dynamic properties and the immersion of the patient into the virtual scenario are significantly improved. There are two types of virtual tools offered by CHAI3D: single interaction point and interdependent points. Dexterous manipulation in the proposed virtual scenario is achieved by the multi-point contact tools, imitating grasping movement via a coupled virtual gripper.

In the designed virtual scenario, physical properties of the virtual objects, such as size, dimension and weight, can be flexibly configured. The virtual braces for the training tasks can also be customized with respect to the position, orientation and relative distance to achieve alternative difficulties for intuitive and adaptive complementary task implementation. Thus, the subjects are kept challenged and actively engaged in the training sessions, and a patient-tailored and neurocognitive therapy has been implemented, where the performance outcomes can be exported for further analysis.

### 2.2. Paradigm of the Bilateral Rehabilitation System

**Figure 3:**
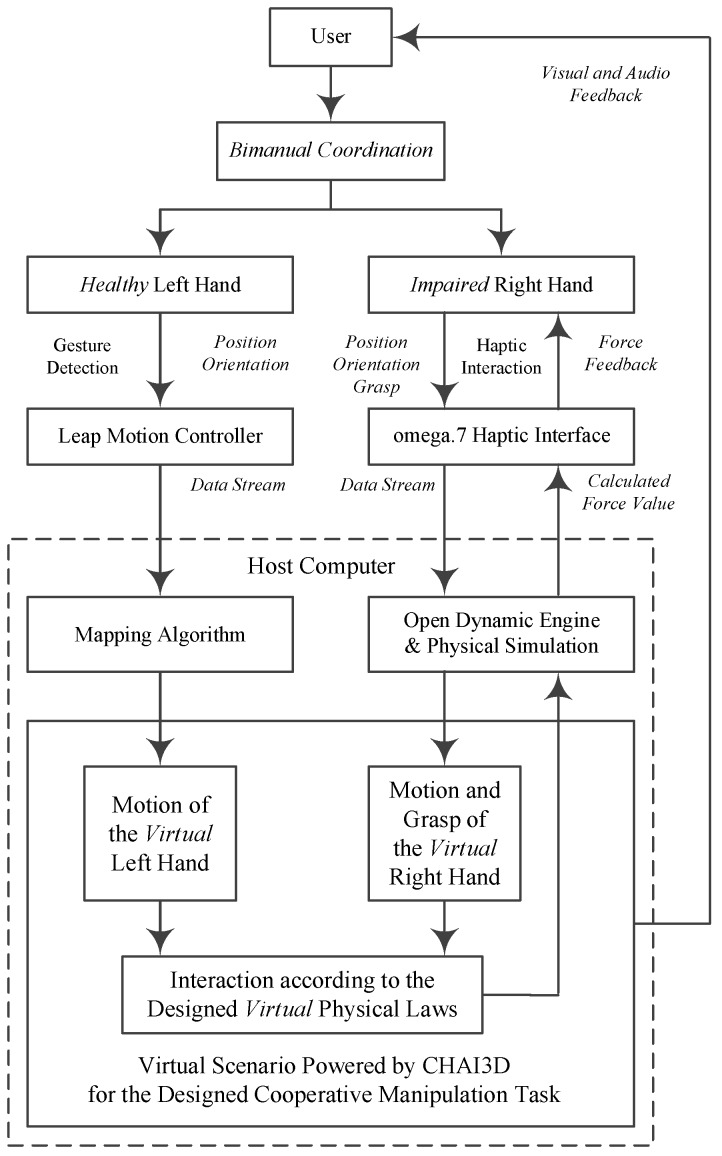
The paradigm of the bilateral arm rehabilitation system.

The paradigm of the proposed bilateral arm rehabilitation method is shown in Figure 3. It allows bilateral complementary tasks for the training of the coordinated cooperation of the paretic arm and intact arm. Without loss of generality, it is assumed in the training session that the user’s left arm is healthy and detected by the Leap Motion sensor, while the right arm is impaired, assisted by the haptic sensor omega.7. The data stream goes into the host computer via USB cable connections. The proposed virtual scenario for the designed bilateral complementary tasks is implemented, powered by the CHAI3D haptic engine. Assistive force feedback is rendered and interacts with the subject, along with essential visual and audio feedback.

**Figure 4:**
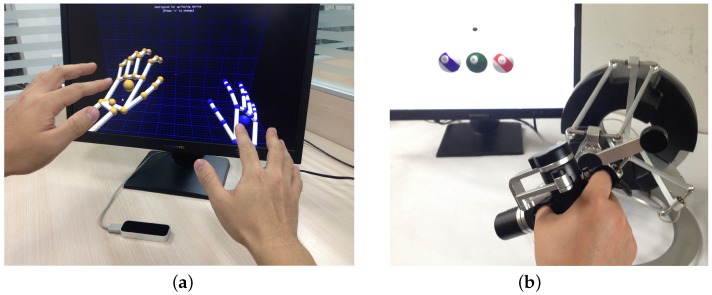
(**a**): the Leap Motion sensor tracks the motion of a healthy hand; (**b**): the omega.7 device assists the impaired hand with force feedback.

The Leap Motion sensor has been taken to track the gesture of the healthy supportive hand. With its declared 200-fps tracking frame rate and sub-millimeter accuracy, this device provides an easy solution for hand motion detection, as shown in Figure 4. The Leap Motion sensor operates in an intimate proximity, recognizes and tracks the motions of hands, fingers and finger-like tools and reports discrete positions, gestures and motion. The evaluation of the accuracy and robustness of the Leap Motion sensor [29] has shown an average accuracy of 0.7 mm. Axis-independent deviation under experimental conditions between the desired static 3D position and measured positions is below 0.2 mm. For dynamic setups, the average accuracy is 1.2 mm, independent of the plane. The repeatability error is less than 0.17 mm. Thus, the Leap Motion device offers a portable, compact economic and reasonably precise sensor solution to realize various human-machine interaction applications in the field of AAL (ambient assisted living) and ADLs [30,31,32], especially for the elderly and disabled users.

The omega.7 device, as shown in Figure 4, introduces an advanced haptic sensing interface with high stiffness due to its parallel kinematics structure. It has overall 7 degrees of freedom (DOFs) movement capabilities, of which 3 DOFs are for rotation, 3 DOFs for translation and 1 DOF for grasp or grip. The 4 DOFs’ precise force feedback is provided during the active translational and grasping movements [30]. The movement range for grasping is 25 mm, with a resolution of 0.006 mm, and the force feedback is up to ±8.0 N, enabling dexterous manipulation and haptic interaction [4,31]. The workspace for translational movements is Φ160 × 110 mm^3^, with a resolution of better than 0.01 mm, and the force feedback can go up to 12.0 N. The rotational workspace of the omega.7 device is 240 × 140 × 180 in degrees, with a resolution of 0.09 degrees. The close-loop stiffness offered by the interface is up to 14.5 N/mm.

With such an implementation, the system is compact, easy to install/uninstall and transfer. Besides, omega.7 is an end-effector-based device. The force and movement ranges are limited, and the patient can easily detach the hand, which provides inherent safety. Considering both the ease of use and inherent safety, no special personnel is mandatory for the application of our system. Thus, it suits convenient use at home, as well as in centralized rehabilitation training institutions.

### 2.3. Fusion of the Haptic and Gesture Sensors and Algorithms

**Figure 5:**
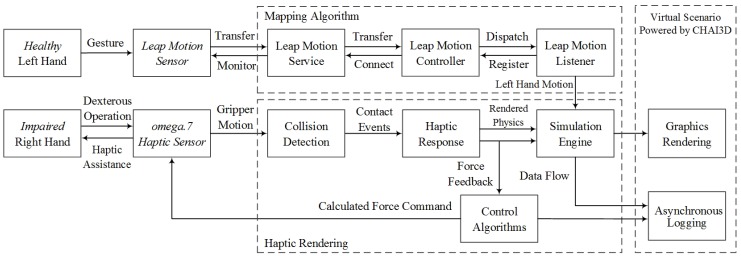
The fusion scheme for the gesture and haptic sensors and the supporting algorithms.

In the proposed bilateral rehabilitation system, the haptic and gesture sensors, together with the haptic rendering and visual-audio feedback algorithms, must be fused together to work synchronously and smoothly with each other, as illustrated in Figure 5.

The Leap Motion sensor that tracks the intact left hand and the omega.7 that haptically interacts with the impaired right hand operate in two coordinate systems. The first step is to implement rigid body transformation to link the two coordinate systems. The reference system of the Leap Motion sensor is a right-handed Cartesian coordinate system. Positive values of the vertical *y*-axis increase upwards. The *x*- and *z*-axes lie horizontally, with the *x*-axis parallel to the the edge of the device. In the virtual scenario simulated by CHAI3D, the *x*-axis and *z*-axis are in the same direction as the *z*-axis and *y*-axis of the Leap Motion sensor, respectively. Given the CHAI3D coordinate system, the Leap Motion sensor coordinate system and the rotation matrix, denoted as {*A*}, {*B*} and *R*(*θ*), respectively, the homogeneous transformation is calculated as:

(1) PA1=RθBAPBORGA01PB1,andRθBA=010001100

When the user’s left hand is detected by the Leap Motion sensor, the discrete position and orientation of the palm are recorded and processed via the listener and controller module, as shown in Figure 5, imported from the programming interface of the Leap Motion sensor. Due to the inconsistent coordinate origin between the Listener module and virtual scenario, discrete positions are processed by a digital differentiator, and relative translations of left palm are interpreted to map the trajectory of the virtual plate and then transited to the simulation engine.

Furthermore, tracking performance under dynamic scenarios reveals inconsistent sampling frequency [33], and the practical tracking frame rate is less than the required high haptic thread (up to 1000 Hz) and graphic rendering (approximately 30 Hz). This sampling inconsistency brings dramatic difficulty to synchronize the communication thread, and unnatural movements may occur in the virtual scenario. To generate natural and steady movements for the virtual hand, a linear interpolation method is applied in the mapping algorithm, interpreting relative translation between the left hand and virtual plate. When the collision detectors engage the attachment between the virtual hand and virtual plate, the interpolation process is activated. The time interval from the previous discrete tracking frame is calculated as *t_in_*, while the interval of the output is calculated as *t_out_*. Thus, the rendering translation of the virtual plate is calculated as:

(2) $$\left[\begin{array}{c} x_{plate}\\ y_{plate}\\ z_{plate} \end{array}\right]=\frac{t_{out}}{t_{in}}\cdot \left(\left[\begin{array}{c} x_{LHand\_next}\\ y_{LHand\_next}\\ z_{LHand\_next} \end{array}\right]-\left[\begin{array}{c} x_{LHand\_current}\\ y_{LHand\_current}\\ z_{LHand\_current} \end{array}\right]\right)$$

The user’s right hand operations are applied on the virtual gripper via the omega.7 haptic device. When the position, orientation and grasp movements are detected by the 7-DOF interface, they are interpolated to map the virtual trajectory of the coupled gripper. As shown in Figure 3, through the physical simulation procedures, the translational and rotational motion of the virtual gripper are then generated as:

(3) $$\left[\begin{array}{c} x_{grp}\\ y_{grp}\\ z_{grp} \end{array}\right]=\left[\begin{array}{ccc} k_{x} & & \\ & k_{y} & \\ & & k_{z} \end{array}\right]\left[\begin{array}{c} x_{opr}\\ y_{opr}\\ z_{opr} \end{array}\right],\left[\begin{array}{c} \alpha_{grp}\\ \beta_{grp}\\ \gamma_{grp} \end{array}\right]=\left[\begin{array}{ccc} k_{\alpha } & & \\ & k_{\beta } & \\ & & k_{\gamma } \end{array}\right]\left[\begin{array}{c} \alpha_{opr}\\ \beta_{opr}\\ \gamma_{opr} \end{array}\right]$$

Here, the parameters *k_x_*, *k_y_*, *k_z_*, *k_α_*, *k_β_*, *k_γ_* can be flexibly configured to achieve a natural dexterous manipulation of the gripper, while the active involvement of the users can be specifically adjusted. Thus, the subjects are kept challenged and actively engaged in the training.

For the proposed complementary task, assistive force feedback is crucial for the immersion and engagement of the user in the virtual environment. When the contact events of collision detection are positive, the virtual interaction force is simulated by the Open Dynamic simulation engine, as shown in Figure 5. During the grasp operation, alternative force feedback generated from the coupled virtual gripper, calculated by the impedance control algorithm, is applied to the omega.7 interface. In the proposed system, 6-DOF haptic rendering is implemented according to the dexterous manipulation operation [34]. Based on a spring-damper coupling between the virtual gripper and dynamic objects, the actual operation force guides the virtual gripper to apply an opposite virtual force on dynamic objects. Rendered force is proportional to the spring displacement, while the torque is proportional to the orientational difference. Given the force and torque value denoted as *F_spring_*, ${\overset{⇀}{\tau }}_{spring}$, respectively, the precise haptic feedback, which requires fine motor control capabilities to manipulate, is rendered as:

(4) $$\begin{array}{ccc} F_{spring}=k_{T}x+b_{T}v, & & {\overset{⇀}{\tau }}_{spring}=k_{R}\overset{⇀}{\theta }+b_{R}\overset{⇀}{\omega } \end{array}$$

where *k_T_*, *b_T_* represent the stiffness and viscosity in spring translation, *k_R_*, *b_R_* represent the stiffness and viscosity in spring rotation and *v* and $\overset{⇀}{\omega }$ describe the relative linear and angular velocity of the dynamic object, respectively. Combining the active manipulation and assistive force feedback, the complementary training of the coordinated cooperation of the paretic arm and intact arm is implemented in the designed scenario.

### 2.4. Data Protocol and Asynchronous Logging

To validate the designed bilateral rehabilitation system and assess the efficacy of the training diagram, parameter data with respect to the task phases should be exported and stored for further analysis, such as the grip force, grip angle and movements of virtual objects.

**Figure 6:**
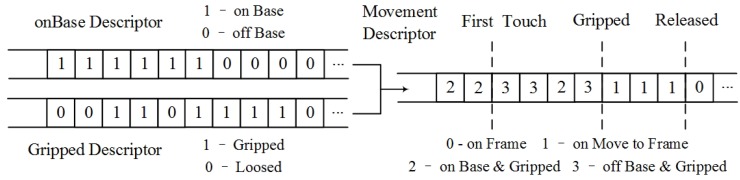
The bit strings for movement description.

In the proposed data processing procedure, bit-vector arrays are utilized to describe the movements and status of the virtual gripper and objects. As shown in Figure 6, the descriptor *d_touch_* (on-base descriptor) indicates whether the object is on the virtual plate, while *d_grip_* (gripped descriptor) illustrates whether the object is gripped.

The descriptors are assembled by performing comparisons of virtual positions, rendered by the previously mentioned scheme. Each bit of *b_touch_* and *b_grip_* can be generated as:

(5) $$\begin{array}{ccc} b_{touch} & = & \left\{\begin{array}{cc} 1 & \left|z_{obj}-z_{plt}\right|\leq 0.5\cdot \left(d_{obj}+d_{plt}\right)\\ 0 & otherwise \end{array}\right. \end{array}$$

(6) $$\begin{array}{ccc} b_{grip} & = & \left\{\begin{array}{cc} 1 & I\left(pos_{obj},pos_{plt}\right)>5\\ 0 & otherwise \end{array}\right. \end{array}$$

(7) $$\begin{array}{ccc} b_{index} & = & 2^{1}\cdot b_{touch}+2^{0}\cdot b_{grip}\; \end{array}$$

where *z_obj_* and *z_plt_* represent the position of the virtual object and gripper in the *z*-axis, respectively, and *d_obj_* and *d_plt_* represent the physical parameters of the virtual object and gripper in depth, respectively. If the virtual object is attached on the plate, the *b_touch_* is positive. The embed function *I*(*pos_obj_*, *pos_plt_*) calculates the amount of interaction points in any contact events. When the number exceeds a predefined threshold value, the *b_grip_* is positive. As illustrated in Figure 6 and Equation (7), the bit-string for movement description is generated by combining the on-base descriptor and gripped descriptor in a binary to decimal conversion manner. Each value in bit *b_index_* describes the current status of the virtual object.

To clearly distinguish the four phases of the operation (as shown in Figure 1 and Figure 2), three flags are defined, *first touch*, *gripped* and *released*. The time stamp for the first occurrence of each flag is logged. As illustrated in Figure 6, the virtual object may slip out of the gripper and need to be gripped more than once, bringing confusion in the movement descriptor data. With these three flags, each phase of the entire operation can be strictly distinguished from each other, and the reliability of the corresponding data can be ensured.

**Figure 7:**
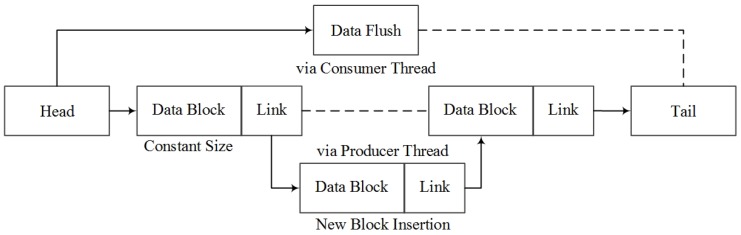
The blocked linked list structure for data logging.

The haptic rendering thread must avoid notable discretization artifacts. On the other hand, the disk I/O operation can be of high latency of up to 10 ms. Under this race condition, the haptic update rate may go lower than 1000 Hz, and the haptic performance may become unstable [35]. To efficiently record the data stream and parameters for further analysis, simultaneously ensuring haptic rendering quality, an asynchronous logging thread is designed and implemented. As shown in Figure 7, the logging thread utilizes a blocked linked list for the data structure. Each node in the list is replaced by a data block with constant size, which enables random insertion via the haptic (producer) thread.

**Figure 8:**
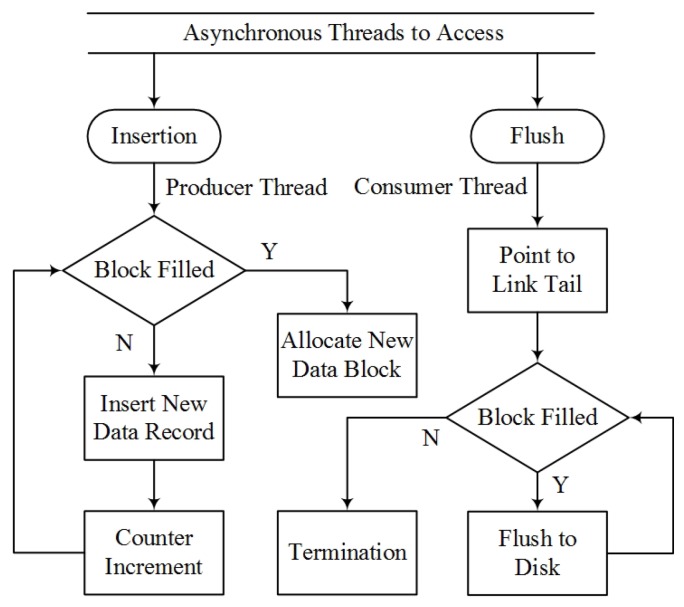
The lock-free data synchronization scheme.

To eliminate the potential conflicts during logging, the head pointer of this hybrid data structure is exclusive to the logging (consumer) thread, and the tail pointer is accessed by the producer thread only. Based on the blocked linked list data structure, a lock-free data synchronization scheme is designed and implemented, as shown in Figure 8. The insertion function for the new data stream is callable exclusively from the producer.

When the data block is full, a new block will be allocated for insertion, and the consumer thread may access the block afterwards. When the consumer thread is engaged, all accessible data can be flushed to the disk, except for the case that the block is not filled up. The constant size of each data block can be adjusted to balance the computing efficiency and cost between the producer thread and consumer thread. A size of 100 has been used in the presented system. Together with the asynchronous logging scheme, this effectively separates the producer and consumer thread and eliminates the race condition.

## 3. Experiments

### 3.1. Subjects

Initial experiments have been conducted to validate the designed bimanual rehabilitation platform and methods, to assess learning effects and to compare the unilateral and bilateral training. A total of 17 young healthy subjects (2 female, 15 male), aged from 21 to 26, consented to participate in the preliminary experiments. All subjects showed right hand dominance following the Edinburgh-handedness inventory. No ethic approval is required.

### 3.2. The Warm-up Session

**Figure 9:**
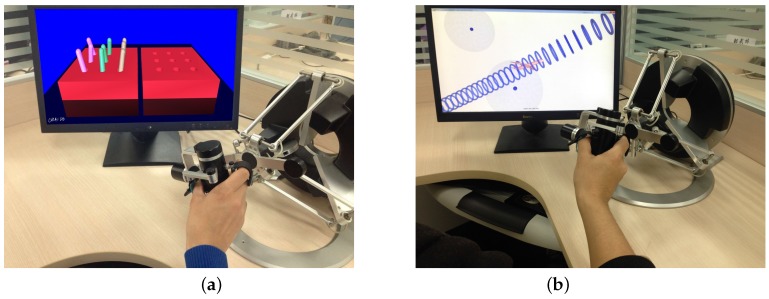
Warming-up exercises with (**a**) the *nine peg insertion* virtual scenario; and (**b**) the *flying through a spatial pathway* virtual scenario.

**Figure 10:**
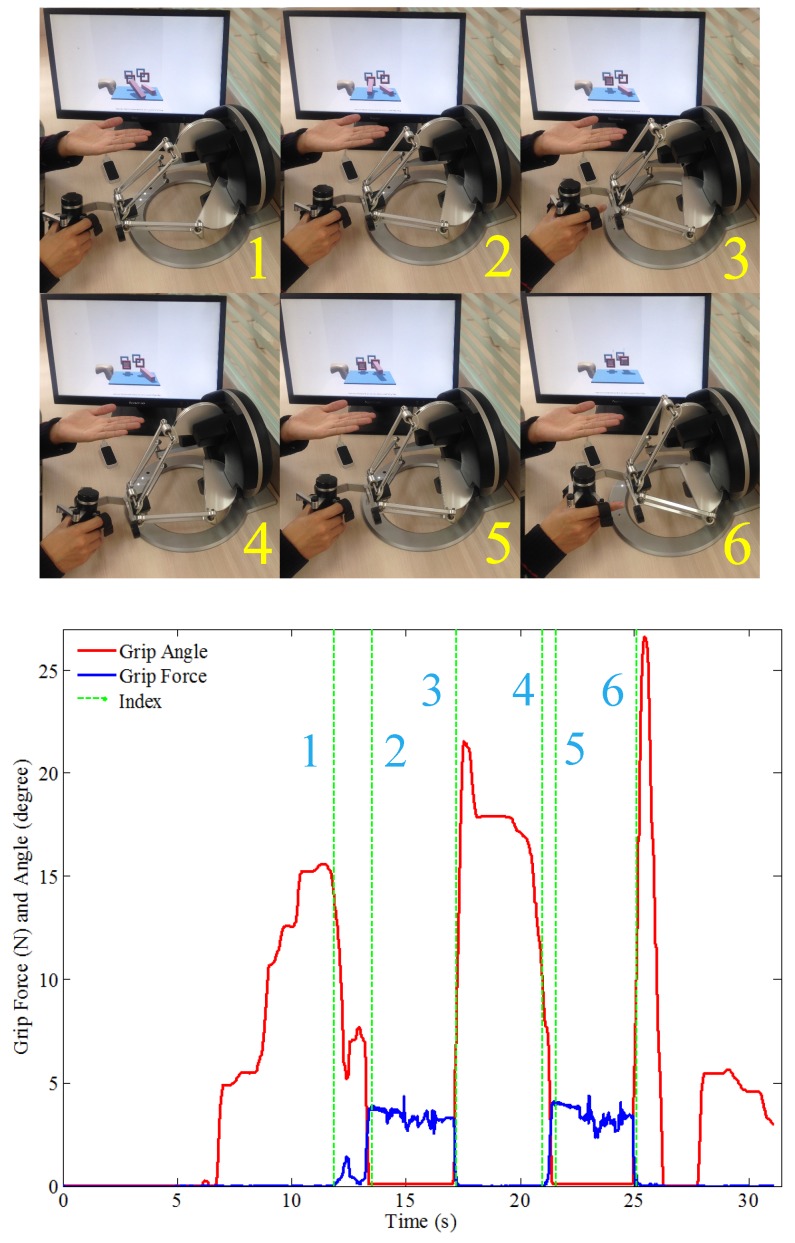
Procedures of each operation. Before 1: the hand is detached; the grip force is about zero; and the gripper angle is uncontrolled. Between 1 and 2: the user grips the first block; the grip force grows; and the gripper angle decreases. Between 2 and 3: the user moves the first block to the target brace; the grip force persists; and the gripper angle remains low. Between 3 and 4: the first block is in place; the gripper moves to the second block; the grip force is low; and the gripper is opened for the second grip. Between 4, 5 and 6: repeats 1, 2 and 3 for the second block. After 6: operation accomplished; no grip force; and the gripper angle is uncontrolled.

As shown in Figure 9 and Figure 10, the designed experimental task consists of the unilateral warming-up session and the bilateral complementary task training session. In the warming-up session, subjects were asked to conduct the *“nine peg insertion”* task for two trials and the *“flying through a spatial pathway”* virtual scenario for one trial, which are unilateral training tasks developed in our previous work [4]. The virtual peg insertion test helps subjects get familiar with the haptic device and virtual scenarios, while specific performance parameters derived from kinematic data can be selected and analyzed to assess and determine the sensory deficits and motor impairment of the subjects [36]. Subjects were instructed to manipulate the virtual gripper to grasp, move and insert the specific pegs into nine holes with their right hands. Certain active forces are required from subjects to accomplish the task.

The *“flying through a spatial pathway”* virtual scenario helps subjects get familiarized with assistive force feedback via the omega.7 interface. With altered gravitational force and disturbance force fields, subjects were instructed to move the aircraft avatar from the starting position to the final position through the designed tunnel, which was generated by a smooth trajectory. Visual and haptic information is integrated to guide the subjects to complete the dexterous training. Active physical and cognitive involvements, such as stabilizing the avatar against gravitational and disturbance force fields, keep the subjects challenged during the familiarization session [37,38,39].

### 3.3. Bilateral Training Tasks

The subjects are engaged with the designed virtual scenario, as shown in Figure 2 and Figure 10, to complete the bilateral complementary task through active coordination and dexterous manipulation. The user’s right hand, representing the paretic side, manipulates the handle of the omega.7 device. The user’s left hand, representing the intact side, holding and moving the virtual plate through the Leap Motion sensor, is complementary to the dexterous manipulation of the right hand. The goal of the training task is to put the specific virtual blocks onto the corresponding virtual braces steadily and smoothly.

As shown in Figure 10, each operation of the proposed training can be divided into seven phases. When the virtual plate is moved by the left hand to the ambient space of the right hand, the virtual gripper approaches and touches a virtual block for the first time. The grip angle tends to decrease while certain grip force is applied to the block, during the interval between 1 and 2. Then, between 2 and 3, this block is moved to the target position, with the grip force persisting, and the gripper angle remains low. When the first block is put on the target frame, the subject opens the gripper again, approaching the second block. Then, the operation is conducted with the second block between 4, 5 and 6, just like with the first block. In the implemented complementary task, the left hand is taken as the non-dominant healthy side, to support and lead the paretic arm by moving and holding the virtual plate. The left hand offers supporting and stabilizing functionality. Precise manipulation is achieved by the arm, especially the wrist and forearm movements, in series with the grip.

### 3.4. Experimental Design

**Experiment 1 for the Technical Validation of the System and Methods** 

Six healthy subjects (1 female, 5 male, age 23.8 ± 0.8) performed the tasks as shown in the previous subsection. A 2 × 2 factorial design was implemented with different friction coefficients of the virtual environment (low/high) and density of virtual blocks (low/high). To reject experimental design bias, seven trials in each of the 4 experimental conditions were randomized and tested for the subjects.

**Experiment 2 for the Assessment of Learning Effects** 

A customized set of experiments was conducted to evaluate the learning progress in the proposed bilateral complementary training session. Three healthy subjects (1 female, 2 male, aged 22.3 ± 1.2) were instructed to perform six trials each day for five consecutive days. A short break of 10 minutes was taken before each trial. The experimental condition was consistent with the low friction coefficient and the low density of virtual objects.

**Experiment 3 for the Comparison of Unilateral and Bilateral Training** 

This experiment was to explore the differences between unilateral and bilateral rehabilitation methods in the proposed virtual scenario. Eight healthy subjects (all males, aged from 21 to 26) were randomly divided into two groups. They were instructed to conduct the customized experiment for 14 sessions. The independent variable is the operation approach in the training session: unilateral or bilateral. The first group performed the task only with the dominant hand, with their supportive hands constrained. Subjects were instructed to grip the virtual blocks and to put on the corresponding virtual braces unilaterally, while the virtual plate remains at a constant location. For the second group, subjects were instructed to perform the complementary task in a coordinated bilateral manner, as illustrated in Figure 10. Participants were blinded to the study hypotheses.

## 4. Results

Task execution time, motion trajectories of all virtual objects, rotational motion of the virtual gripper (angles of pitch, yaw and roll), path length of the left and right hands, along with the grip force and angle were recorded and exported for initial analysis of the training experiments, especially for the assessment of dexterous manipulation.

For the parameters of mean grip force, maximum grip angle, traveled path length, execution time and index of coordination, specific analysis of variance (ANOVA) was performed to analyze the effects of experimental parameters on performance across all experimental conditions. The threshold of significance was set to 0.05. When the ANOVA yielded a significant difference, Tukey’s honestly significant difference test was performed.

### 4.1. Results of Experiment 1: Technical Validation of the System and Methods

**Figure 11:**
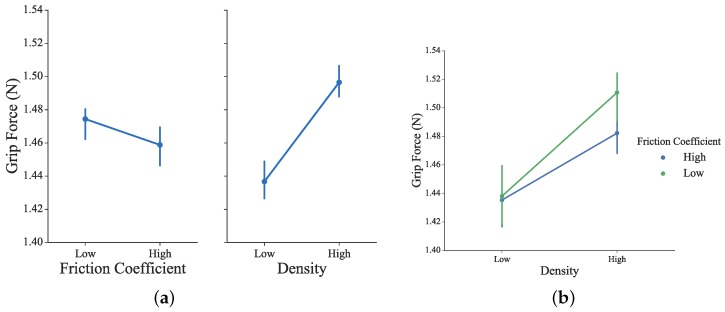
(**a**) Mean grip force of all subjects under four experimental conditions; (**b**) Mean grip force of all subjects across all conditions in the factor plot.

The grip strength represents the actual operation force applied on the omega.7 haptic sensor. It has been calculated from the active physical force of the right hand and the rendered assistive force feedback of the virtual scenario. As shown in Figure 11, to grasp the virtual block with alternative conditional parameters, necessary grip force is generated from the right hand. Slightly but not significant differences were observed under different conditions of the blocks’ density and friction coefficient values (for density: *F*_1156_ = 1.01, *p* = 0.32; for friction coefficient: *F*_1156_ = 0.07, *p* = 0.79). The higher the density is or the lower the friction is, stronger grip force is required from the subjects. Thus, fine motor control capabilities of grip force by the paretic side are required and trained.

**Figure 12:**
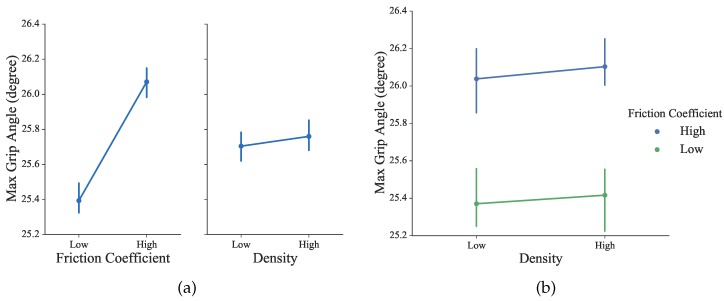
(**a**) Max grip angle of all subjects under four experimental conditions; (**b**) Max grip angle of all subjects across all conditions in the factor plot.

The changes in the parameter of grip aperture, which is the distance between the index and the thumb finger, have been taken to characterize grasping by Jeannerod [40]. In our proposed training session, the grip angle calculated from the coupled virtual grippers can be used to equivalently describe the changes in grip aperture, because the subjects’ thumb and index fingers are rigidly bounded with the omega.7 stylus. As shown in Figure 10, during a typical grasp movement, it was observed that the gradually increasing aperture reached its maximum values at about 60–70% of the duration, following by shrinking until fully grasped. As shown in Figure 12, differences were observed under different conditions, but not statistically significant (for density: *F*_1156_ = 0.01, *p* = 0.91; for the friction coefficient: *F*_1156_ = 2.07, *p* = 0.15). This is consistent with previous observations that the object size, weight and texture of the contact surface can influence the kinematics of grasping [41].

**Figure 13:**
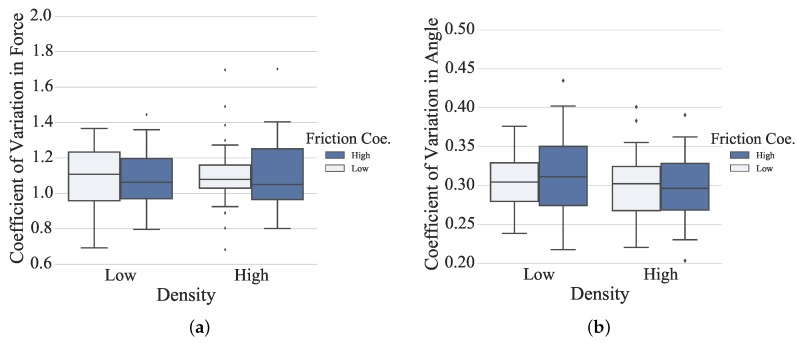
(**a**) The coefficient of variation of mean grip force across all conditions in box plot; (**b**) The coefficient of variation of max grip angle across all conditions in box plot.

An adapted difficulty level of rehabilitation therapy is essential for extensive engagement and minor frustration [42]. The coefficient of variation (CV) can be an effective measure to evaluate the challenging level of the proposed rehabilitation task to distinguish the performance in each experimental conditions [43]. The higher CV value suggests that the experimental condition is more challenging. As shown in Figure 13, from the grip force point of view, no significant differences were observed under different conditions (for density: *F*_1156_ = 0.59, *p* = 0.44; for the friction coefficient: *F*_1156_ = 0.04, *p* = 0.82). The highest CV value was obtained for the condition of low density under low friction, which can be challenging to the sensorimotor system in the sense that the smallest grip force needs be produced and fine force control capability is required. The CV values in the other three conditions were very close. From the grip angle point of view, no significant differences were observed under different conditions (for density: *F*_1156_ = 2.53, *p* = 0.11; for the friction coefficient: *F*_1156_ = 0.05, *p* = 0.83). It is not clear yet whether the maximum grip aperture indicates the key point of the kinematic duration or a preliminary portrait of the grasp, especially in the virtual scenario, where the perception of objects is different from the physical world.

**Figure 14:**
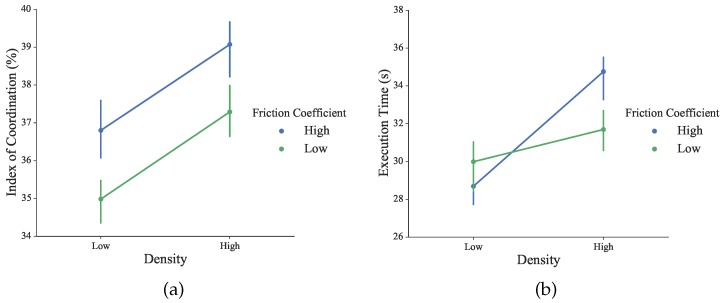
(**a**) The index of coordination of all subjects across all conditions in the factor plot; (**b**) Execution time of all subjects across all conditions in the factor plot.

To efficiently measure the subject’s performance of bilateral coordination in the proposed complementary tasks, the index of coordination (IC) is defined as:

(8) $$IC=\frac{L\left(\left|P_{LHand}P_{grp}\right|<d_{thr}\right)}{L(P_{grp})}\cdot 100\%\;$$

where *P_LHand_* and *P_grp_* are respectively the three-dimensional position of the left hand and the gripper that is operated by the right hand, in the virtual world. The predefined threshold value *d_thr_* is introduced to distinguish the close distance |*P_LHand_P_grp_*|, which is between the subject’s two hands. The embedded function *L*(*x*) returns numbers of the specific elements. Thus, the IC measures the proportion of the execution time where both hands are in the same performable ambient space to cooperate, rather than to operate separately. As shown in Figure 14, slightly but not significant differences were observed under different conditions (for density: *F*_1156_ = 1.19, *p* = 0.28; for the friction coefficient: *F*_1156_ = 0.73, *p* = 0.39). Higher IC values indicate that the user tends to perform the complementary task with more bimanual cooperation.

The execution time across all conditions was calculated to assess the bilateral performance during the training session. As shown in Figure 14, blocks with high density required more time to finish. Across all of the conditions, differences of execution time were not statistically significant (for density: *F*_1156_ = 1.32, *p* = 0.25; for the friction coefficient: *F*_1156_ = 0.07, *p* = 0.79).

**Figure 15:**
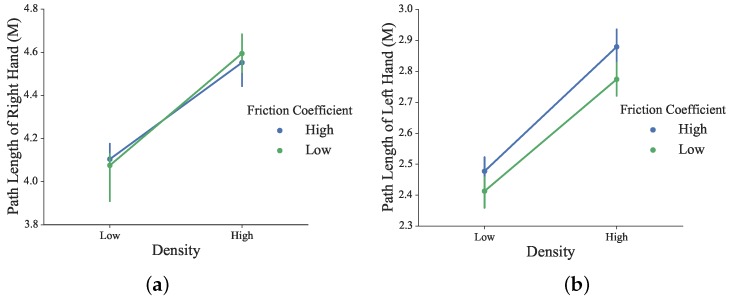
(**a**) Path length of the right hand; (**b**) Path length of the left hand during the task.

The traveled path length of the left and right hands during the task is shown in Figure 15. For both the right and left hand, the higher the density, the longer the traveled path was. The effect of the friction coefficient was weak. The statistical test showed no significant difference in the path length of the right under different conditions of the blocks’ density and friction coefficient values (*F*_1156_ = 2.65, *p* = 0.11 for density; *F*_1156_ = 0.0005, *p* = 0.98 for the friction coefficient). Significant differences in the path length of left hand were observed under different conditions of the blocks’ density (*F*_1156_ = 4.37, *p* = 0.04). *Post hoc* analysis showed the statistical difference in path length between different densities. Both the supportive left hand and manipulative right hand have traveled a longer path for the high density condition.

### 4.2. Results of Experiment 2: Learning Effects

**Figure 16:**
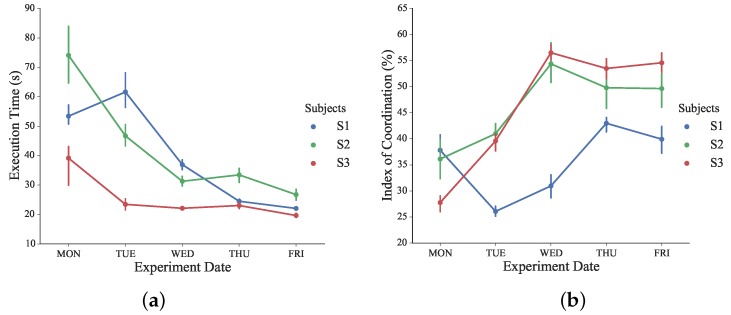
(**a**) Execution time of all subjects during the learning phase; (**b**) The index of coordination of all subjects during the learning phase.

The evolution of execution time and the IC index during the designed learning experiments is shown in Figure 16. A significant difference in the execution time was observed during the consecutive days of learning sessions (*F*_475_ = 9.90, *p* < 0.05). *Post hoc* analysis revealed the statistical difference in execution time since Day 3 (Wednesday). The execution time of all subjects declined from Day 2 (Tuesday) to Day 3 (Wednesday). As a spatiotemporal parameter of assessment, a smaller time window to complete tasks indicates that better performances have been obtained after two days of training sessions [42].

No significant difference was found in multiple comparisons within the last three days. From the execution time point of view, the performances of all subjects evolved in a steady manner after sufficient training sessions. A significant difference in IC was observed during the five days of training sessions for all subjects (*F*_475_ = 5.56, *p* < 0.05). *Post hoc* analysis revealed a statistical difference in the index of coordination since Wednesday.

Significant improvements in IC were achieved between Tuesday and Wednesday for all subjects. The proposed IC measures the subject’s performance of bilateral coordination in a temporal-spatial manner, and higher IC values indicates that the involvement of bimanual coordinated cooperation increased gradually and voluntarily.

No significant difference was obtained in multiple comparisons within the last three experiment days. After previous training sessions, subjects became more familiar and proficient with the bilateral complementary task training. Higher and steadier IC values were achieved through more voluntary and coordinated bimanual cooperation. Thus, complementary bilateral functional skills were re-trained and assessed during the proposed consecutive training sessions.

### 4.3. Results of Experiment 3: Comparison of Unilateral and Bilateral Training

**Figure 17:**
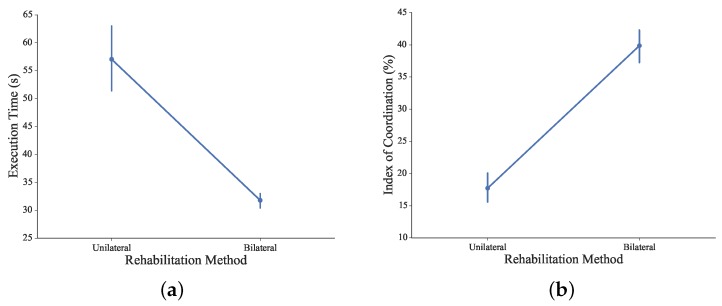
(**a)** Comparison of execution time between the unilateral and bilateral methods; (**b**) Comparison of the index of coordination between the unilateral and bilateral methods.

The comparison of execution time and IC between unilateral and bilateral methods is shown in Figure 17. With the unilateral rehabilitation method, subjects’ left hands were constrained, and their right hands performed the complementary task solely.

Without the stabilization and support from non-dominant hands, significant differences in the execution time were observed via different approaches of the rehabilitation method (*F*_1110_ = 37.41, *p* < 0.05). *Post hoc* analysis showed the statistical difference in execution time between the different methods. With the coordinated cooperation from both arms, the execution time was significantly decreased to accomplish the proposed bilateral complementary task.

From the IC point of view, significant differences were observed via different approaches of the rehabilitation method (*F*_1110_ = 116.25, *p* < 0.05). *Post hoc* analysis showed the statistical difference in the index of coordination between different methods. The IC increased significantly in bilateral training, indicating that bimanual coordination were extensively and voluntarily involved, promising a complementary re-training of fine motor control capabilities via the proposed rehabilitation system.

## 5. Conclusions and Outlook

In this work, a novel bilateral arm rehabilitation method has been designed and implemented with the fusion of the Leap Motion gesture sensor and omega.7 haptic sensor. Complementary bilateral skills that are mostly required in ADLs are re-trained through the designed bilateral tasks, encouraging coordinated cooperation of upper extremities and dexterous manipulation of the impaired hand. A virtual scenario involving visual, audio and assistive force feedback has been built up. With this approach, motor, sensory and cognitive processes are closed in one loop and trained all together. Preliminary experiments have been successfully conducted with healthy subjects, showing good technical feasibility for rehabilitation training and assessment at home or clinical institutions. Initial analysis has been performed with the recorded data, and the results are consistent with the literature for several outcome measures. Besides, the proposed method has enabled and inspired further research potentials.

Future work will be two-fold. Functional outcome measures need to be developed for the bimanually-coupled temporal-spatial data, which is not available yet for cooperative and manipulative tasks that are initiated and dominated by the patient. Clinical study with stroke patients are being planned.
